# The close relationship between heparanase and epithelial mesenchymal transition in gastric signet-ring cell adenocarcinoma

**DOI:** 10.18632/oncotarget.26042

**Published:** 2018-09-18

**Authors:** Shahid Shah, Caroline Fourgeaud, Simon Derieux, Shahsoltan Mirshahi, Geneviève Contant, Cynthia Pimpie, Rea Lo Dico, Jeannette Soria, Marc Pocard, Massoud Mirshahi

**Affiliations:** ^1^ Lariboisière Hospital, INSERM U965, Sorbonne University Paris Cité -Paris 7, 75010 Paris, France; ^2^ Diagnostica Stago, Gennevilliers 92230, France; ^3^ Present address: Department of Pharmacy Practice, Faculty of Pharmaceutical Sciences, Government College University, Faisalabad, Pakistan

**Keywords:** carcinomatosis, epithelial mesenchymal transition, heparanase, KATO-III cell line, signet ring cell adenocarcinoma

## Abstract

Heparanase (HPSE), a heparan sulfate-specific endo-β-D-glucuronidase, plays an important role in tumor cell metastasis through the degradation of extracellular matrix heparan sulfate proteoglycans. Suramin, a polysulfonated naphthylurea, is an inhibitor of HPSE with suramin analogues. Our objective was to analyze the HPSE involvement in gastric signet ring cell adenocarcinoma (SRCA) invasion. High expression of HPSE mRNA and protein was found in the tumor and in ascites of SRCA as well as in KATO-III cell line. Beside of collagen-I, growth factors (TGF-β1 and VEGF-A, except FGF-2) and epithelial mesenchymal transition (EMT) markers (Snail, Slug, Vimentin, α-SMA and Fibronectin, except E-cadherin) were found higher in main nodules of SRCA as compared to peritumoral sites. Among MDR proteins, MDR-1 and LRP (lung resistance protein) were highly expressed in tumor cells. The formation of 3D cell spheroids was found to be correlated with their origin (adherent or non-adherent KATO-III). After treatment of KATO-III cells with a HPSE inhibitor (suramin), cell proliferation and EMT-related markers, besides collagen-1 expression, were down regulated. In conclusion, in SRCA, HPSE via an autocrine secretion is involved in acquisition of mesenchymal phenotype and tumor cell malignancy. Therefore, HPSE could be an interesting pharmacological target for the treatment of SRCA.

## INTRODUCTION

Gastric signet ring cell adenocarcinoma (SRCA) is characterized by the presence of isolated or small groups of malignant non-cohesive cells (>50%) containing intracytoplasmic mucin and exhibits diffuse growth and invasion without forming ducts [1]. Peritoneal invasion is the most frequent type of metastasis in patients with SRCA. It frequently occurs at the later stages of gastric carcinoma, especially after surgery and significantly contributes to gastric cancer-related mortality [2]. One of the characteristics of SRCA is its resistance to chemotherapy. Most tumor cells with multidrug resistance are characterized by the over-expression of multidrug resistance molecules such as P-glycoprotein (P-gp), lung resistance protein (LRP) and multidrug resistance-associated protein (MRP) [3, 4]. Another mechanism of chemoresistance is the epithelial-mesenchymal transition (EMT) [5]. EMT plays an essential role in tumor development namely cancer metastasis [6] and fibrosis [7]. During EMT, cells display on one hand, decreased expression of epithelial cell markers such as E-cadherin and on the other hand, increased expression of mesenchymal cell markers (Snail, Slug, Vimentin, α-SMA, fibronectin, collagen-I) and enhanced cell motility [8, 9]. SRCA was also characterized by the presence of tissues fibrosis [10]. EMT associated with hypersecretion of heparanase (HPSE) was reported in renal fibrosis. HPSE as a multitasking protein, characterized by enzymatic and non-enzymatic activities, modulates TGF-β-induced EMT and fibrosis [11, 12]. Enzymatically active HPSE binds to the cation-independent mannose 6-phosphate receptor (CD222) expressed on cell surfaces to degrade extracellular matrix [13] with heparan sulfate, a side chain of heparin sulfate proteoglycans (HSPGs) [14]. HSPGs store various cytokines and growth factors such as basic fibroblast growth factor-2 (FGF-2), vascular endothelial growth factor (VEGF), interferon-β (INF-β), and transforming growth factor-β (TGF-β) [15–17]. HPSE has also non-enzymatic functions [18]. The signaling activity is achieved by interacting with transmembrane proteins, leading to an activation of Akt and Src [19], or modulating the activity of factors such as FGF-2 and TGF-β [12, 20]. Recent analysis showed that degradation of heparan sulfate by HPSE increased permeability across the basal membrane and, thus, stimulated the release of cytokines and growth factors leading to tumor development via angiogenesis and metastasis [15, 17, 21]. Recently, protein or messenger RNA (mRNA) expression of HPSE has been identified in various cancer cells and the over-expression of HPSE protein or mRNA in tumor cells has been reported and correlated with the metastatic potential of tumor cells *in vitro* and *in vivo* as well as with poor prognosis [22, 23]. However, its role in SRCA is still not clearly clarified.

Suramin, a polysulfonated naphthylurea, was initially used to treat African parasitic infections, such as Rhodesian and Gambian trypanosomiasis [24]. Suramin, an inhibitor of HPSE and its analogues showed antiangiogenic and antiproliferative properties [25]. Increased levels of circulating glycosaminoglycans have been observed in suramin-treated cancer patients, suggesting that it might inhibit glycosaminoglycan catabolism [26]. Suramin has also been shown to inhibit HPSE in many human cancer cell lines by independent groups [27]. Despite the U.S. Food and Drug Administration disapproving the use of suramin at therapeutic concentrations, low or non-cytotoxic doses of suramin might be used as effective treatment in SRCA.

The aim of this study was to identify the position of HPSE in the SRCA malignancy and its inhibition by suramin.

## RESULTS

### Gastric signet ring cell adenocarcinoma nodules express HPSE

**Figure 1:**
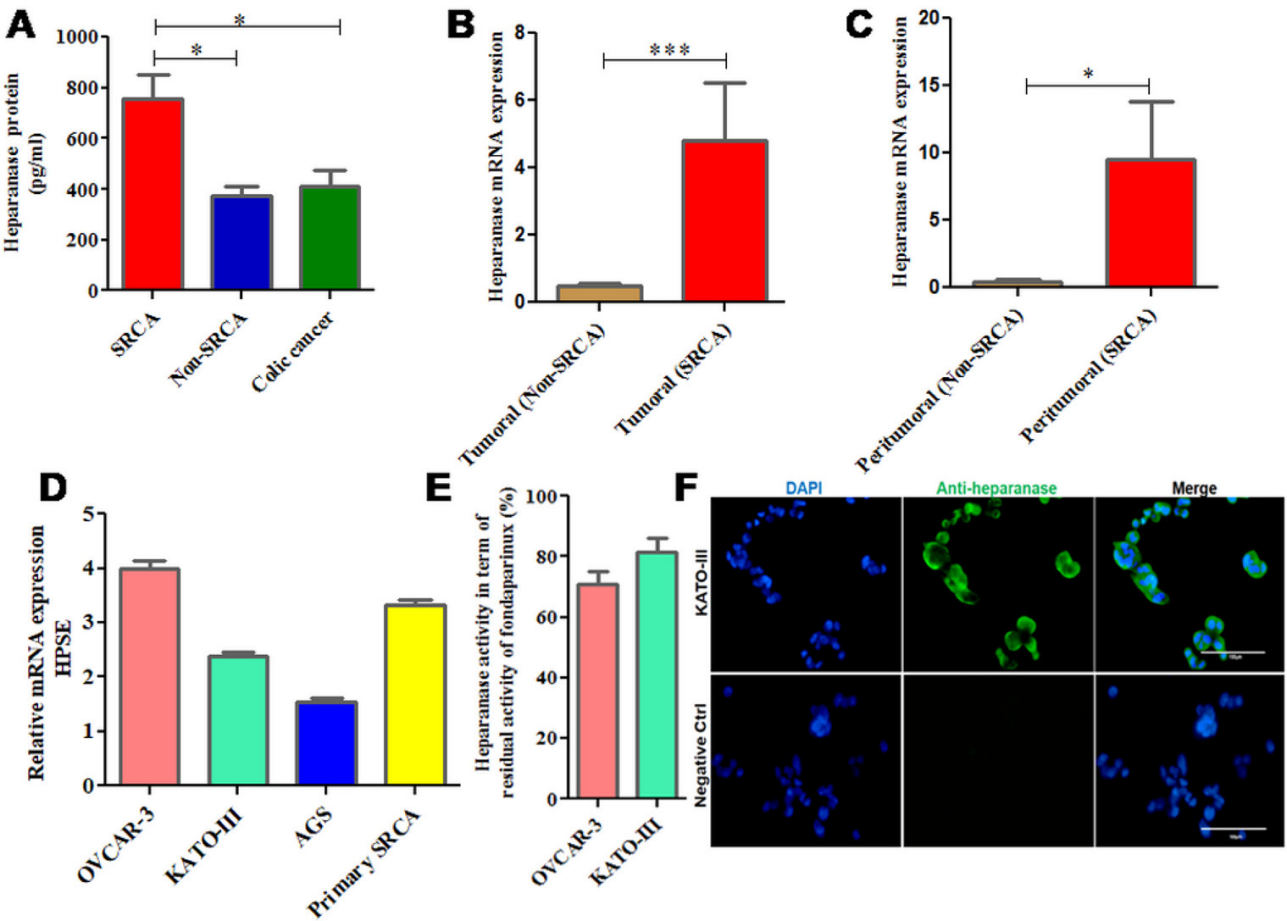
mRNA and protein expression of heparanase in clinical samples and cell lines including KATO-III Heparanase protein was found in the ascitic samples of primary signet ring cell adenocarcinoma (SRCA) of stomach as compared to Non-SRCA of stomach and colic cancer by ELISA (SRCA *n =* 5, Non-SRCA *n =* 3 and colic cancer *n =* 6) (**A**) mRNA expression of heparanase was found higher in SRCA (*n =* 11) than non-SRCA (*n =* 10) (**B**) as well as in peritumoral-SRCA (*n =* 7) than peritumoral non-SRCA (*n =* 8) (**C**) Heparanase gene expressed by various cell lines ovarian (OVCAR-3), gastric (AGS, KATO-III), and primary SRCA (Primary GC) via RT-PCR, (**D**) Heparanase activity (evaluated by degradation of fondaparinux at pH 5) observed in supernatants of various cancer cell lines (OVCAR-3) and gastric (KATO-III), (**E**) Heparanase protein expression level in KATO-III by immunofluorescence is shown (**F**) The results are expressed as mean ± SEM of three independent experiments ^*^P < 0.05, ^***^P < 0.001, statistically significant.

As presented in Figure 1A, high expression (*p = 0.0327*) of HPSE protein was found in the ascitic samples of primary gastric signet ring cell adenocarcinoma (SRCA) as compared to gastric non-SRCA and colic cancer by ELISA. Figure 1B shows that mRNA expression of HPSE is significantly higher in the tumoral zone of gastric SRCA than non-SRCA (*p = 0.0002*). HPSE mRNA was also found to be highly expressed in peritumoral zone of SRCA. As presented in Figure 1C, HPSE mRNA was found to be more (*p* = 0.015) in peritumoral-SRCA as compared to peritumoral non-SRCA. Relative expression of HPSE by several cancer cells such as primary SRCA cells isolated from peritoneal fluid of SRCA patients and various cell lines such as ovarian (OVCAR-3), gastric (AGS, KATO-III) presented in Figure 1D. Relative HPSE activity from the supernatants of ovarian (OVCAR-3) and gastric (KATO-III) cell lines presented in Figure 1E. The presence of HPSE was confirmed by immunohistochemistry in KATO-III cell line (Figure 1F). These results are in favor of high expression of HPSE mRNA and protein in gastric SRCA as found by qPCR and ELISA respectively in SRCA.

### Gastric signet ring cell adenocarcinoma express EMT and multi drug resistance markers

**Figure 2:**
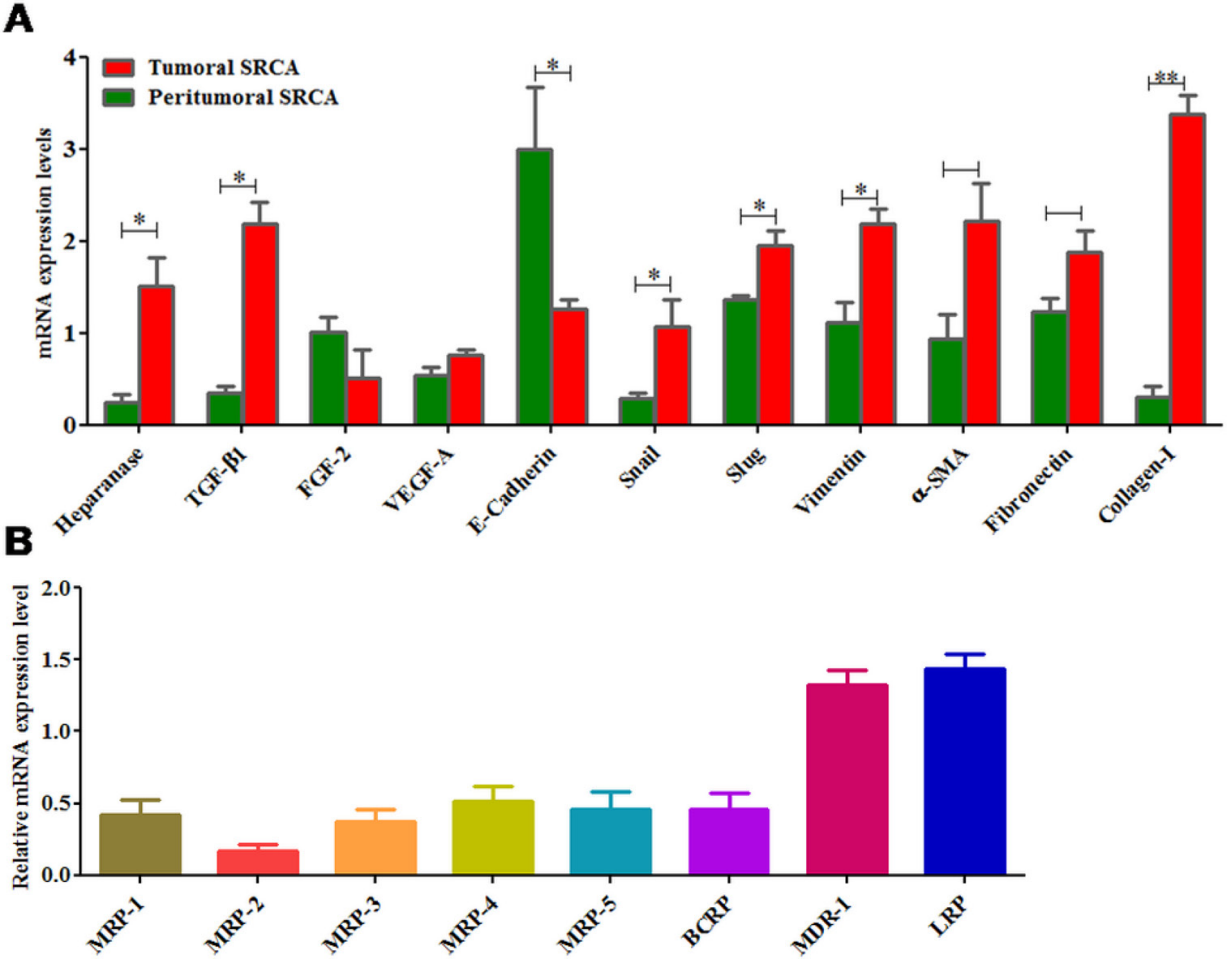
mRNA expression of heparanase, growth factors, EMT markers and drug transporters in clinical samples Heparanase, growth factors (TGF-β1 and VEGF-A) except FGF-2 and epithelial marker like E-cadherin were found higher while mesenchymal markers (Snail, Slug, Vimentin, α-SMA and fibronectin) were lower in tumoral tissue of SRCA as compared to peritumoral tissue by qPCR (**A**). Similarly, of all the drug transporters (MDR-1, MDR-2, MDR-3, MDR-4, MDR-5, BCRP, MDR-1 and LRP) only two (MDR-1 and LRP) were found higher in SRCA tissue samples (**B**). The results are expressed as mean ± SEM of six independent experiments ^*^P < 0.05, ^**^P < 0.01, statistically significant.

Figure 2A presents the relative mRNA expression of several growth factors such as FGF-2, TGF-β1 and VEGF-A as well as EMT markers (E-cadherin, Snail, Slug, Vimentin, α-SMA and fibronectin) in tumoral tissues of SRCA as compared to the peripheral region of tumor. Except E-Cadherin and FGF-2, all other markers tested were expressed highly in tumor tissues as compared to periphery of tumors. These results suggest mesenchymal characteristics of tumor tissues and are also in favor of drug resistance in SRCA’s patients. When the drug transporter (MDR-1 (Pg-1), MDR-2, MDR-3, MDR-4, MDR-5, BCRP, MDR-1 and LRP) mRNA expression in tumor region, determined by RT-qPCR, as presented in Figure 2B, all ATP-binding cassette proteins as well as lung resistant protein were detected. However, among these drug transporters, MDR-1 and LRP were the most expressed.

### KATO-III cell line formed spheroid clusters and expressed EMT markers *in vitro*

**Figure 3:**
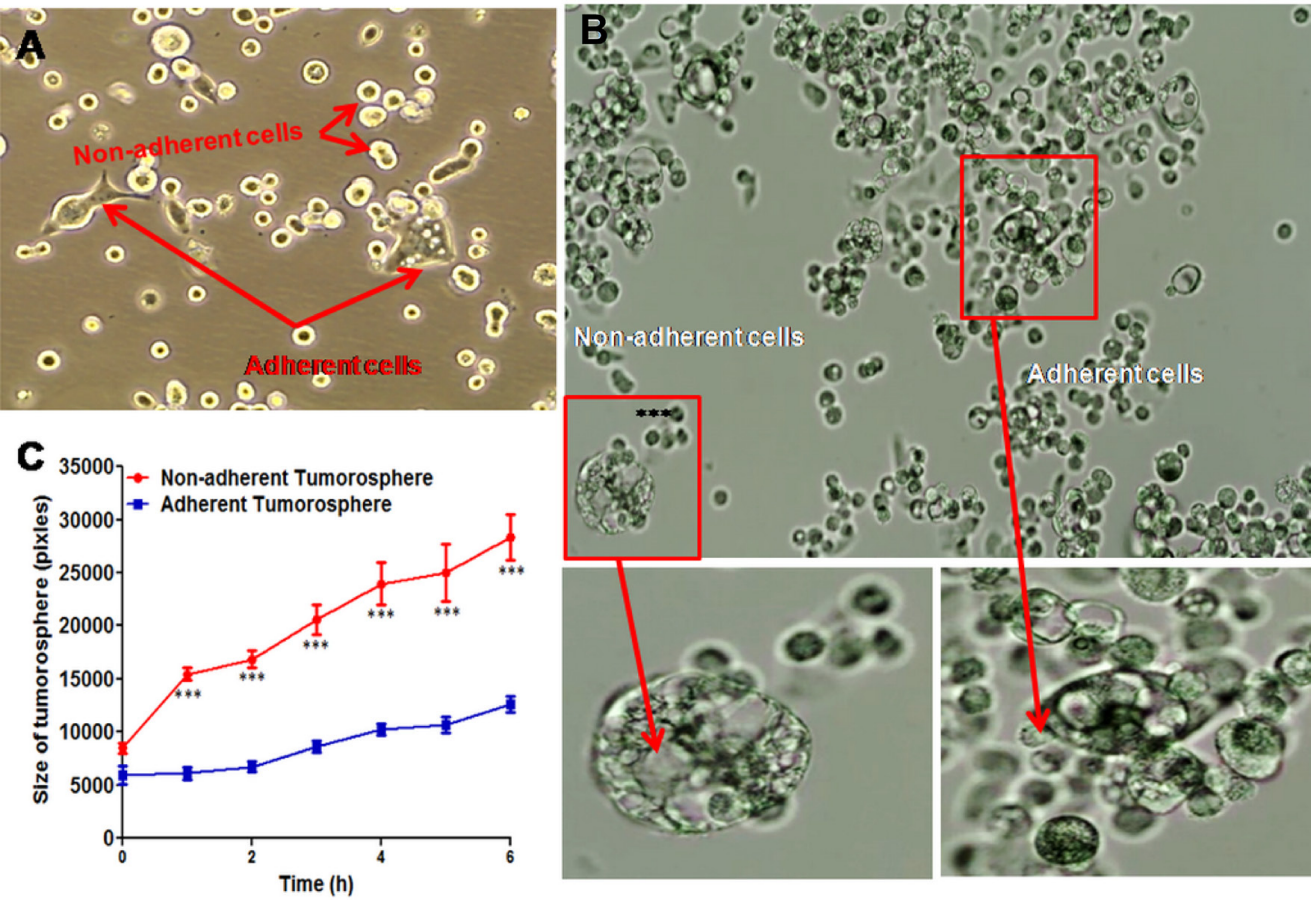
Spheroid cluster formation as well as cytokine array of both adherent and non-adherent KATO-III cell line Adherent and non-adherent cells of KATOIII (**A**) Tumorosphere cells derived from adherent and non-adherent KATO-III (**B**) quantification of the maximal tumorosphere outgrowth diameter (pixels) during 6 hrs (**C**).

In culture medium, KATO-III cell line has two phenotypes; adherent and non-adherent cells (Figure 3A) and formed the spheroids. As presented in Figure 3B, micro-cinematographic studies of these cell clusters indicated that their diameters varied according to their origin (adherent and non-adherent cells). As presented in Figure 3C, the non-adherent cells are more spherogenic than adherent cells (*p = 0.0001*). Gene expression of EMT markers in both adherent and non-adherent KATO-III cells was studied. No alteration in gene expression (*p = 0.124*) of epithelial-mesenchymal transition (EMT)-related molecules was observed when adherent and non-adherent KATO-III cells were grown separately for one week (Supplementary Table 2).

### Suramin, an HPSE inhibitor down regulated TGF-β and collagen-1 mRNA KATO-III cells

**Figure 4:**
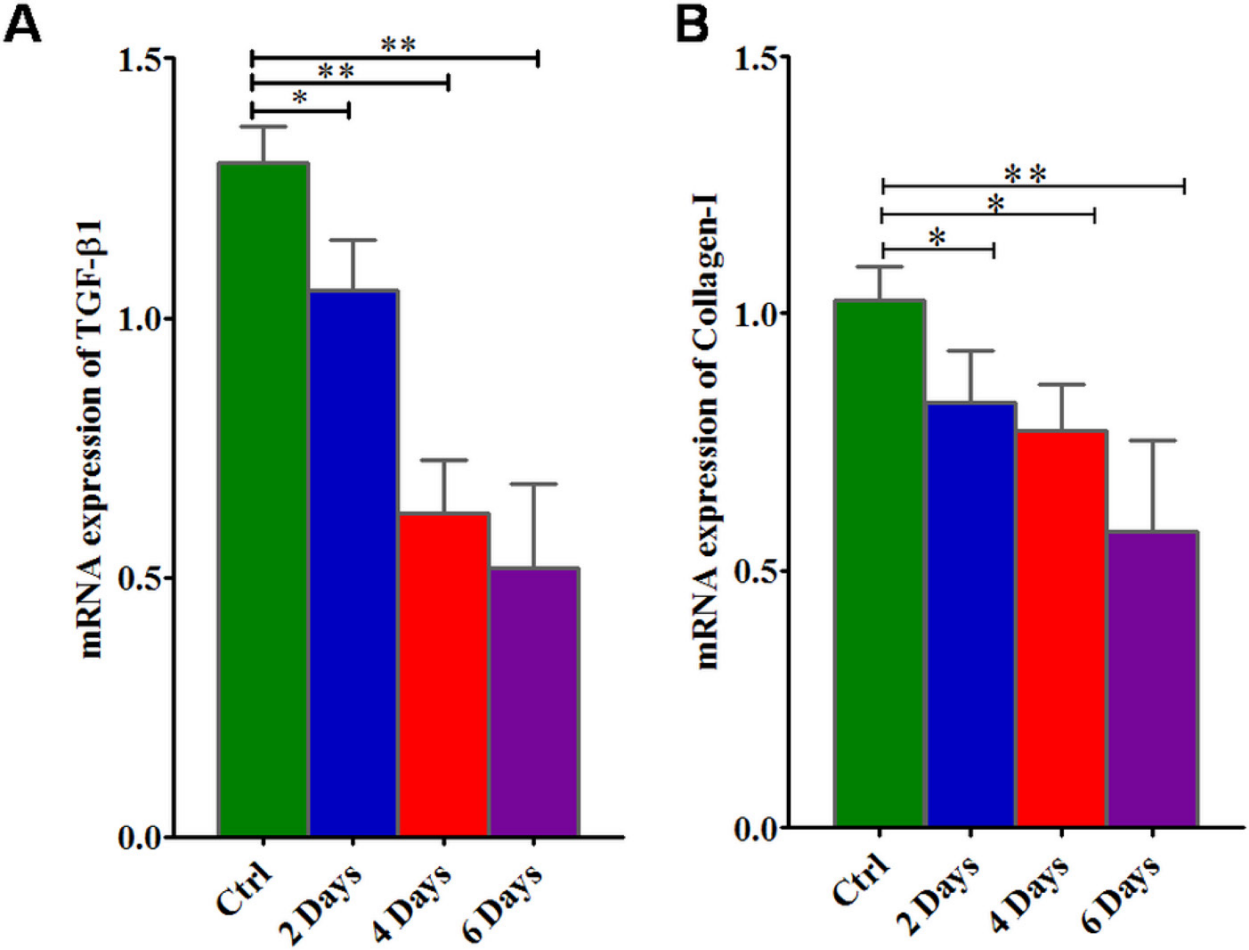
mRNA gene expression of TGFβ-1 and collagen-I in KATO-III cell line after treatment with suramin Suramin lowers the expression of TGFβ-1 (**A**) and collagen-I (**B**) in KATO-III in a time dependent manner by qPCR. The results are expressed as mean ± SEM of six independent experiments ^*^P < 0.05, ^**^P < 0.01, statistically significant.

As presented in Figure 4, when KATO-III cells were cultured in a medium containing suramin, there is a down regulation of TGFβ-1 and collagen-I expression in SRCA cell line in a time dependent manner. This result suggests that suramin simultaneously down regulates TGFβ-1 and collagen-1 expression in KATO-III cells.

### Suramin down regulates EMT and stem cell markers as well as inhibits cell cycle and proliferation of KATO-III cell line

**Figure 5:**
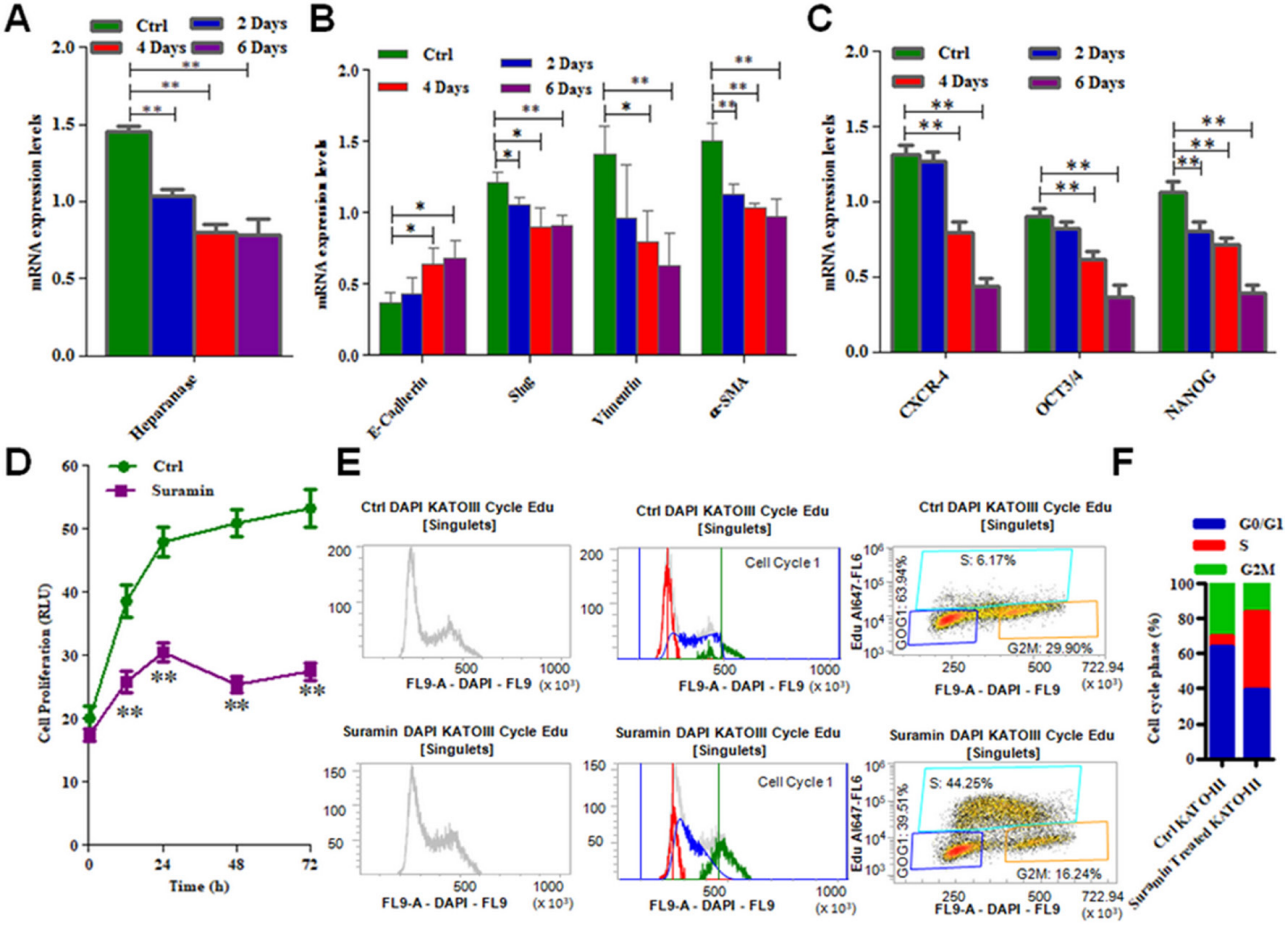
mRNA expression of EMT markers, stem cell markers as well as cell cycle arrest and cell proliferation in KATO-III after treatment with suramin Expression of heparanase (**A**), mesenchymal markers (Slug, Vimentin and α-SMA) (**B**) and stem cell markers (CXCR-4, OCT3/4 and NANOG) (**C**) were found lower while epithelial marker (E-cadherin) was found higher (B) in KATO-III after treatment with suramin by qPCR. Suramin inhibits the proliferation (**D**) and cell cycle arrest (**E**) in KATO-III after treatment. The data indicates the percentage of cells in each phase of the cell cycle (**F**). The results are expressed as mean ± SEM of six independent experiments ^*^P < 0.05, ^**^P < 0.01, and are statistically significant.

As presented in Figure 5A, incubation of KATO-III cell line in a culture medium with suramin, down regulates HPSE expression in SRCA cell line in a time dependent manner. After 6 days incubation of these cells with suramin, mesenchymal markers such as Slug, Vimentin and α-SMA are significantly decreased (*p = 0.005*) while the E-cadherin is up regulated (*p = 0.01*) (Figure 5B). Suramin also decreased (Figure 5C) the expression of stem cell marker CXCR4, OCT3/4 and NANOG (*p = 0.005*). The inhibition of KATO-III cell proliferation was observed when these cells were incubated with suramin (*p = 0.005*) (Figure 5D). The influence of suramin on cell cycle (G1, M and G2) of KATO-III is presented in Figure 5E. The percentage of cells in each phase of the cell cycle is presented in Figure 5F). These results indicate that suramin via phase S pathway inhibits cancer cell proliferation.

### Suramin modified phosphokinase activity pattern of KATO-III cells

As presented above, KATO-III cell line, in an autocrine manner or as a matter of fact by a paracrine pathway, secretes HPSE and influences endogenous kinase activity. The base line of phosphokinase activity of KATO-III cells (mean of three experiments) grown in the serum free culture medium presented is in Supplementary Figure 1A. These results indicate that some phosphokinase pathways such as GSK-3α/β, β-catenin, Chk-2, AMPKα-1, PRAS40 and C-Jun (more expressed proteins presented) are active in these cells. This pattern was modified when the cells were incubated in culture medium with the suramin. Results presented in Supplementary Figure 1B indicate that 6 different kinases were altered in KATO-III treated for 5 hrs as compared to the control. After treatment with suramin, two of the markers were upregulated (HSP60 and C-Jun) whereas 4 (GSK-3α/β, β-catenin, Chk-2 and AMPKα-1), were down regulated. These results correspond to the inhibition of secretion of non-activated HPSE and indicate the crucial role of HPSE in cell hemostasis.

These results so far presented (Figures 1–5 and Supplementary Figure 1) provide data that are important in elucidating the goal of the present work.

## DISCUSSION

In our study, we have demonstrated high level of HPSE in SRCA (both tumor and ascitic fluid) and inhibition of HPSE induced EMT after addition of an inhibitor of HPSE (suramin).

HPSE is a multitasking protein characterized by enzymatic and non-enzymatic activities. Signaling activity of HPSE is achieved by interacting with transmembrane proteins, modulating the activity of factors such as FGF-2 and TGF-β [11].

HPSE expression was found high in the ascitic samples of the patient with SRCA of stomach as compared to that of patients with non-SRCA of stomach and colic cancer. Furthermore, high HPSE mRNA expression was found in primary gastric SRCA cells, KATO-III cell line as well as in an ovarian cancer cell line (OVCAR-3) as compared to gastric non-SRCA (AGS) cell lines. Active HPSE was also analyzed in the supernatants of cell lines including gastric (KATO-III). SRCA tissue of stomach showed more mRNA expression of HPSE, pro-fibrotic markers (TGF-β1, FGF-2), VEGF-A, mesenchymal markers (Slug, Vimentin) and fibrotic markers (fibronectin, α-SMA and Collagen-I) than those present in their corresponding peritumoral sites.

These findings allow concluding that SRCA are mesenchymal cells expressing high levels of HPSE and fibrotic markers. Our results are compatible with the report of David *et al.* who found HPSE association with intestinal fibrosis *in vivo* [28] and that of Mosala *et al.*, who found FGF-2 dependent EMT related fibrosis in diabetic nephropathy [20]. Our results are also in accordance with the findings reported by the two authors cited previously.

FGF-2 was found absent in both forms of KATO-III cells (adherent and non-adherent) and in low amount from the tumoral tissues of patients with SRCA of stomach as compared to higher amount from peritumoral areas, suggesting its effect emanating from peritumoral sites. The interference of FGF-2 with HPSE [29] as well as TGF-β [30] and IGF-2 [31] for CD222 activity in the tumor microenvironments may modulate epithelial-mesenchymal interactions [20]. We also observed high mRNA expression of IGF2 in gastric SRCA tumoral tissue than peritumoral tissues (*p =* 0.004) as well as gastric non-SRCA (*p =* 0.007) (results not shown). It also affects TGF-β [32] and FGF-2 [33] related functions and inhibits multilineage differentiation of mesenchymal cells [34] as well as promotes tissue fibrosis, cancer expansion and malignancy.

Multi-drug resistance (MDR) remains a great obstacle to effective chemotherapy for SRCA gastric cancer [35]. The present work also indicates, for the first time that in SRCA tumoral cells, LRP and MDR-1 proteins expression is higher than other resistance proteins (MRP1–5, BCRP).

We showed also that KATO-III cells were capable of conversion between two distinct forms (adherent and non-adherent cells), a transition attributed to a reversible adaptive plasticity. We found no alteration in gene expression of epithelial-mesenchymal transition (EMT)-related molecules when adherent and non-adherent cells were grown separately for one week. This finding is consistent with the observation of Jun-Jun She *et al.* who found no difference in tumorigenicity *in vivo* when “*side population*” and “*non-side population*” of KATO-III cells were injected subcutaneously in nude mice [36].

In addition, we observed spheroid clusters of KATO-III that originated from both adherent and non-adherent cells. Size of these spheroid clusters that originated from non-adherent cells was found to be significantly larger than those that originated from adherent cells. Multiple evidences suggested the existence of spheroid clusters after isolation of stem cells from a variety of normal and tumor tissues [37–39] indicating spheroid formation, a common growth characteristic of SRCA cell line. LRP and MDR-1 expression by SRCA tumor, an EMT characteristic of this tumor, indicated the chemoresistance of SRCA patients to treatments.

Clinical trials at non-cytotoxic suramin levels in combination with chemotherapeutic agents have been conducted to treat metastatic breast cancer [40] and advanced non-small cell lung cancer [41], with discernible antitumor activity being noted in the latter. After finding HPSE mRNA and protein in KATO-III cells, we treated these cells with non-cytotoxic dose of suramin (200 µM) and observed increased phosphorylation of HSP60 and c-Jun while GSK-3α/β, β-catenin, Chk-2 and AMPkα-1 decreased. These results suggest the crucial role of HPSE in KATO-III cell homeostasis. GSK-3 has been associated with tumor progression by stabilizing components of the β-catenin complex [42]. Chk-2 as a kinase is involved in cell differentiation [43] and may influence SRCA differentiation and cell fibrosis while activation of AMPkα-1 acts to maintain cellular energy stores, switching on catabolic pathways that produce ATP for cell homeostasis [44]. C-jun phosphorylation is required for maintaining sufficient cyclin D1 kinase activity and for allowing cell cycle progression and apoptosis [45, 46].

Our interest was to focus on the regulation of EMT and fibrosis by altering the expression of EMT markers as well as TGF-β and collagen-I expression respectively using suramin. This was investigated in KATO-III cells in which we showed significant inhibitory effect of suramin on HPSE and collagen-I related fibrosis. Our findings demonstrate that suramin exerts a considerable degree of inhibition on EMT and proliferation in KATO-III cells. Similarly, suramin also induced a G0/G1 cell cycle block of up to 39% and inhibited S phase up to 98% of the cell population. These findings are similar to those reported by HuaPing Li *et al.* who found inhibition of cell proliferation by suramin in ovarian and cervical cancer by down regulating HPSE expression [47]. These results are in good correlation with our results showing that HPSE regulated EMT is inhibited by suramin, an HPSE inhibitor.

We have demonstrated increased HPSE level in SRCA microenvironment, which is responsible for pro-fibrotic factor dependent EMT leading to cell malignancy and fibrosis. The expression level of LRP and MDR-1 was found higher in SRCA that contributes to the chemoresistance observed in this malignancy. Suramin has been shown to be effective in the prevention and treatment of the EMT correlated with the metastatic propensity of tumor cells. HPSE could therefore be an interesting pharmacological target for the treatment of SRCA.

## MATERIALS AND METHODS

### Materials

#### Cell lines and reagents

Human cancer cell lines used, ovarian (OVCAR-3) and gastric (AGS, KATO-III) were obtained from the American Type Culture Collection (ATCC, Manassas, VA, USA).

Ascitic fluids from 14 cancer patients of the Hospital Lariboisière (Paris, France) were collected. As ascitic fluid evacuation is a part of routine management of patients, only oral consents were obtained from them. Cells from ascitic fluids were pelleted by a short spin at 1000 rpm and supernatants were collected after a 10 min centrifugation.

Drug used in this study was suramin (Sigma Chemical Co, St. Louis, MO, USA).

### Culture

Cells were cultured in RPMI 1640 medium or IMDM medium containing 10% heat-inactivated fetal bovine serum (FBS), 50 ug/ml of streptomycin, 50 IU/ml of penicillin and 2 nM of L-glutamine (Gibco, Saint Aubin, France). Cells were incubated at 37° C in a humidified atmosphere containing 5% CO_2._

### Tissues

Tumor and corresponding normal gastric tissue specimen (SRCA tumoral, SRCA peritumoral, Non-SRCA tumoral and Non-SRCA peritumoral) were obtained from 21 patients with signet ring cell adenocarcinoma from the General and Digestive Tract Surgery Department at Lariboisière Hospital in Paris (France). Informed consent was obtained from each patient prior to surgery. All of the tumor and macroscopically normal gastric tissue samples were obtained at the time of surgery. These tissue samples were rapidly frozen in liquid nitrogen and stored at −80° C until analysis. Tissue samples were histologically confirmed by hematoxylin and eosin staining.

### Evaluation of HPSE by ELISA

After centrifugation of ascitic fluids from 14 cancer patients (SRCA *n =* 5, Non-SRCA *n =* 3, Colic carcinoma *n =* 6) at 1200 rpm for 5 minutes at room temperature, the supernatants were collected. The HPSE was quantified by using the commercially available HepAnalyze™ HPSE ELISA Kit (InSight Biopharmaceuticals Ltd. Rehovot, Israel) according to the manufacturer’s instructions. The results were expressed in pg/ml.

### Immunocytochemistry

KATO-III cells grown on multichamber slides were fixed at 25° C for 15 min with 4% paraformaldehyde in PBS (Phosphate Buffered Saline), and rinsed 3 times with PBS. Cells were permeabilized with 0.3% triton for 15 min and then rinsed 3 times with PBS at 25° C. The slides were incubated for 20 min with 1% (w/v) bovine serum albumin (BSA; Sigma-Aldrich, St. Louis, MO, USA) in PBS to block non- specific binding sites and then with anti HPSE polyclonal (1:50 in 1% BSA; Bioss antibodies, Woburn, USA) overnight at 4° C, protected from light. After washing with PBS, the slides were further incubated with a secondary anti- Ig rabbit antibody coupled to FITC (1/500 in 1% BSA) to maximize coloring, for 1 hour at room temperature

### Fluorometric assays

A substrate based activity assay kit (Kaivogen, Turku, Finland) that determines HPSE activity in term of fondaparinux^®^ degradation was utilized according to the manufacturer’s instructions. Briefly, various cancer cell lines (10^6^ cancer cells) ovarian (OVCAR-3) and gastric (KATO-III) were incubated in serum-free culture medium at 37° C in a humidified atmosphere of 5% CO_2_ for 24 hrs. 15 µl of each supernatant diluted to one half of fondaparinux (50 µg/ml) with 135 µl of buffer pH 5.2 were incubated for 2 hrs at 37° C. HPSE activity in term of residual activity of fondaparinux was then evaluated by adding factor Xa and its substrate in each supernatant mixture using STA Compact Max^®^ (Gennevilliers, France) with excitation at 490 nm and emission at 520 nm. Results are expressed in % age.

### Cell viability assay

Cell viability was assayed by Real Time-Glo™ MT Cell Viability Assay. In brief, cells (3 × 10^3^/well) were seeded on 96-well plates, followed 24 hrs later by treatment with drug (or vehicle control) for 96 hrs. Bioluminescence was measured with the spectrofluorometer SAFAS Xenius XC. Cell viability was expressed as the percentage of absorbance of the drug-treated cells relative to that of the vehicle-treated cells.

### RNA isolation, RT and real-time PCR

Gastric tissue specimens were homogenized with a polytron tissue homogenizer. Total RNA in cells and tissues was extracted using the Qiagen RNeasy Mini Kit (Qiagen, Germany) according to the manufacturer’s instruction. RNA samples were transcribed to cDNA in a 20 µl volume by using the QuantiTect reverse transcription kit (Qiagen).

The thermal cycling comprised of the real time PCR as per following conditions: 95° C for 5 min, followed by 40 cycles (denaturation for 15 sec at 95° C, annealing for 20 sec at 60° C and extension for 20 sec at 72° C). The primer sequences and PCR product size for the target and reference genes are listed in Supplementary Table 1.

mRNA expression levels of different markers were detected by real-time PCR with ß-actin as internal reference, using Mesa Blue qPCR Master Mix Plus for SYBR assay (Eurogentec) on the Mastercycler Realplex2 (Eppendorf).

Relative quantitation was calculated by using the comparative threshold cycle (C_T_) method with realplex software. Mean C_T_ of triplicate measurements was used to calculate ΔC_T_ as the difference in C_T_ for target and internal reference (ß-actin) genes. The difference between the ΔC_T_ of the control experiment (KATO-III) and the ΔC_T_ of each sample were calculated to give ΔΔC_T_. Fold increase in mRNA was calculated by 2^−ΔΔCT^.

The PCR products of cell lines and tissue samples after real-time PCR were electrophoresed by E-Gel Precast Agarose Electrophoresis System.

### Human phosphokinase array

A membrane-based antibody array (R&D Systems, Raffles, China) that determines the relative levels of 45 different human phosphorylated protein kinases was used according to the manufacturer’s instructions. Briefly, equal amounts of cell lysates of KATO-III cell line treated with or without 200 µM Suramin (Sigma Chemical Co, St. Louis, MO, USA) into FBS free IMDM medium along with control for 5 hrs were incubated overnight with the phosphokinase array membrane. The array was washed to remove unbound proteins followed by incubation with a mixture of biotinylated detection antibodies. Streptavidin-HRP and chemiluminescent detection reagents were applied to visualize the signal produced at each capture spot corresponding to the amount of phosphorylated protein bound with densitometry by using a photosensitive film (Kodak, X-OMAT, AR, USA).

### Analysis of cell cycle in KATO-III after treatment with suramin

Apoptosis assay was performed by EdU staining using a Click-iT Plus EdU Alexa Fluor 647 Flow Cytometry Assay Kit (Life Technologies), according to the manufacturer’s protocol. KATO-III cell line was synchronized with IMDM medium having 5% FBS for 24 hrs. The following day, cells were incubated for 4 days with or without 200 µM suramin and then trypsinized. These harvested cells were incubated in culture medium with 15 µM EdU for 2 hrs. After incubation, cells were washed with 1% BSA in PBS and 100 µl of Click-iT fixative was added for 15 minutes at room temperature. After washing, cells were incubated in Click-iT Plus reaction cocktail including fluorescent dye (Alexa Fluor 647 picolyl azide) for 30 minutes. The flow cytometric cell analysis was performed, using a BD LSR II analytical flow cytometer (Becton Dickinson, San Jose, CA). MultiCycle AV (Phoenix Flow Systems) DNA analysis software enabled determination of the phase of cell cycle arrest by comparing percentages of each cell stage between the control and treatment groups (G1, S, G2/M).

## SUPPLEMENTARY MATERIALS FIGURE AND TABLES


