# Supplementary material for: The close relationship between heparanase and epithelial mesenchymal transition in gastric signet-ring cell adenocarcinoma
Source: Oncotarget. 2018 Sep 18;9(73):33778–87. doi: 10.18632/oncotarget.26042 (PMC6173471; doi:10.18632/oncotarget.26042)
Supplement: Supplementary file 1 [file oncotarget-09-33778-s001.pdf]

# The close relationship between heparanase and epithelial mesenchymal transition in gastric signet-ring cell adenocarcinoma

## SUPPLEMENTARY MATERIALS

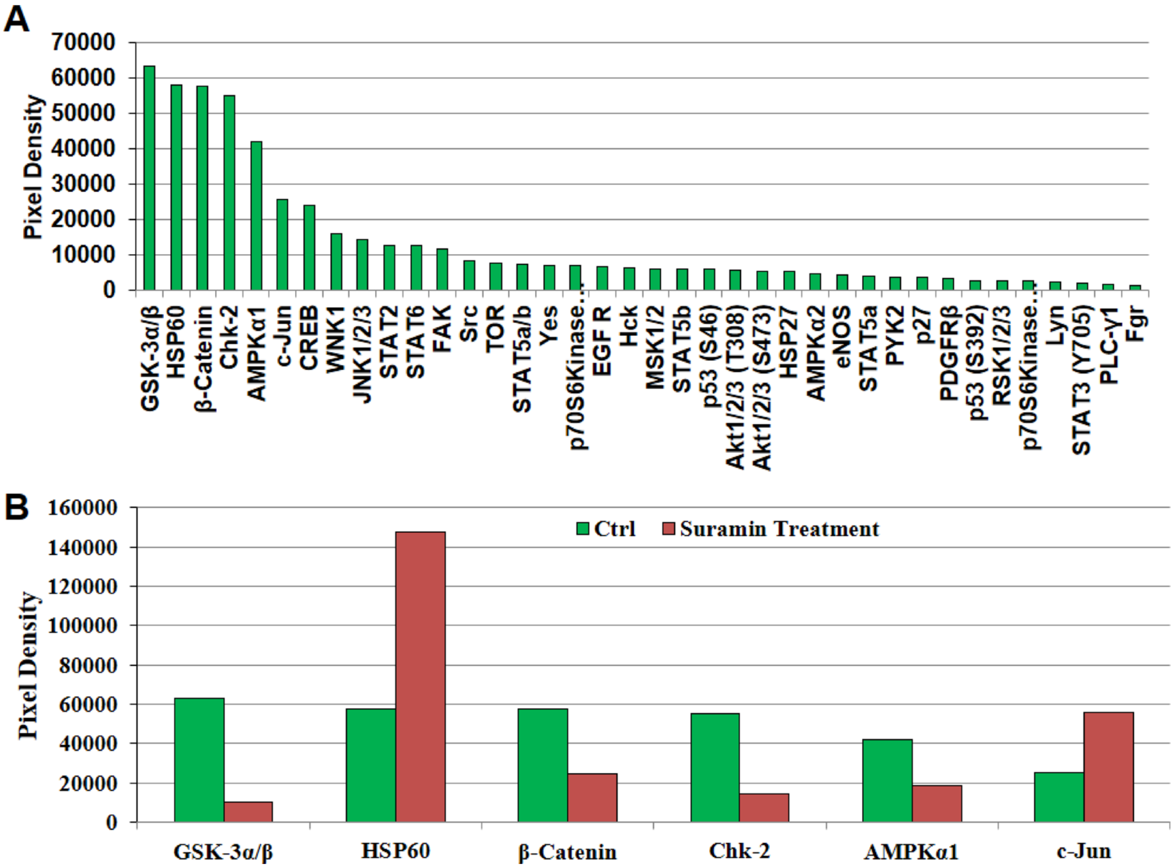


**Supplementary Figure 1: Modification of Phospho-kinase activity in KATO-III cells after treatment with suramin.** 6 different phosphorylated proteins were observed in KATO-III treated with suramin (200 µM) in comparison to the control. 2 of them were up-regulated (HSP60 and C-Jun) while 4 (GSK-3α/β, β-catenin, Chk-2 and AMPkα-1) were down-regulated.

**Supplementary Table 1: Sequence of primers for RT and real-time PCR**

| Gene | Primers (5′→3′) | Product length (bp) |
|---|---|---|
| Hepranase | AGACGGCTAAGATGCTGAAGAG<br>TCTCCTAACCAGACCTTCTTGC | 208 |
| FGF-2 | CTGGCTATGAAGGAAGATGGA<br>TGCCCAGTTCGTTTCAGTG | 149 |
| TGF-β1 | CAGAAATACAGCAACAATTCCTGG<br>TTGCAGTGTGTTATCCCTGCTGTC | 186 |
| VEGF-A | CCCACTGAGGAGTCCAACAT<br>AAATGCTTTCTCCGCTCTGA | 173 |
| E-Cadherin | TGGACAGGGAGGATTTTGAG<br>ACCTGAGGCTTTGGATTCCT | 190 |
| Snail | CCAATCGGAAGCCTAACTACAG<br>GACAGAGTCCCAGATGAGCATT | 155 |
| Slug | GCATTTCTTCACTCCGAAGC<br>TGAATTCCATGCTCTTGCAG | 151 |
| Vimentin | GAGAACTTTGCCGTTGAAGC<br>GCTTCCTGTAGGTGGCAATC | 163 |
| α-SMA | TTCAATGTCCCAGCCATGTA<br>GAAGGAATAGCCACGCTCAG | 222 |
| Collagen-I | CCTGGATGCCATCAAAGTCT<br>AATCCATCGGTCATGCTCTC | 153 |
| Fibronectin | CCGAGGGACCTGGAAGTT<br>ACTTGCTCCCAGGCACAG | 151 |
| MRP-1 | AGGTGGACCTGTTTCGTGAC<br>CCTGTGATCCACCAGAAGGT | 181 |
| MRP-2 | GACCAACATTGTGGCTGTTG<br>GAGGACCAGATCCAGCTCAG | 163 |
| MRP-3 | GGGCGTCTATGCTGCTTTAG<br>CCTTGGAGAAGCAGTTCAGG | 188 |
| MRP-4 | AGAGCTGGTGCTCACTGGAT<br>CGGTTACATTTCCTCCTCCA | 154 |
| MRP-5 | CCTTTTCACTCCCTCCATCA<br>ACAGGTCTTGGAGCTGGAGA | 185 |
| BCRP | CACCTTATTGGCCTCAGGAA<br>CCTGCTTGGAAGGCTCTATG | 200 |
| MDR-1 | TGCCACCACGATAGCTGA<br>CTGCTTCTGCCCACCACT | 172 |
| LRP | GTGGAGGTCGTGGAGATCAT<br>CCAAATCCAGAACCTCCTCA | 186 |
| β-ACTIN | AGAGCTACGAGCTGCCTGAC<br>AGCACTGTGTTGGCGTACAG | 184 |

**Supplementary Table 2: Expression of mRNA of epithelial-mesenchymal transition related markers in adherent and non-adherent KATO III cells**

| mRNA expression ratio | E-Cadherin | Snail | Slug | Vimentin | α-SMA |
|---|---|---|---|---|---|
| **Non-adherent/adherent** | 1.32 | 0.89 | 0.8 | 0.78 | 0.88 |
| **Non-adherent/adherent after grown separately** | 1.24 | 0.89 | 0.9 | 0.81 | 0.75 |

Real-time PCR analysis showed no alteration in gene expression of epithelial-mesenchymal transition (EMT)-related molecules when adherent and non-adherent KATO III cell lines were grown separately for one week.
